# *Sorcerer II:* The Search for Microbial Diversity Roils the Waters

**DOI:** 10.1371/journal.pbio.0050074

**Published:** 2007-03-13

**Authors:** Henry Nicholls

## Abstract

This feature explores bioprospecting and the legal issues related to collecting and cataloging microbial diversity from oceanic locations around the world.

**Figure:**
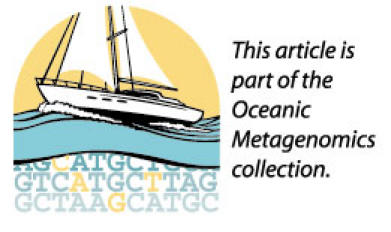


Craig Venter is not short of ambition. With the human genome fresh off the sequencing machines, he set his sights on a project of even grander scale: to describe the immense wealth of genetic information living in the world's oceans. This voyage into biologically uncharted waters was, according to the Web site of the expedition vessel *Sorcerer II*, inspired in part by the voyage of H. M. S. *Beagle* [1]. Venter, it seems, would like to be remembered as the Charles Darwin of the 21st century (Figure 1).

**Figure 1:**
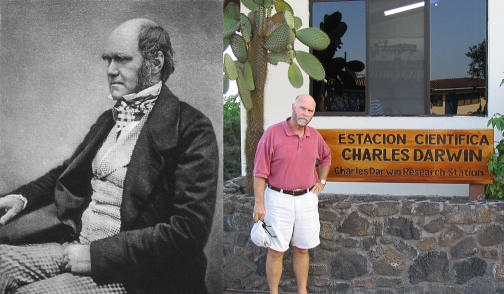
Two of a Kind? The young Charles Darwin (left) and Craig Venter (right). (Photo: J. Craig Venter Institute)

This is the largest effort to describe the genetic diversity in the world's oceans. The voyage around national and international waters, collecting from around 150 sites and interrogating samples at the level of the gene rather than at the level of the organism, has already turned up between 5 and 6 million genes. Most of these genes have never been seen before, says Venter. Analysing this immense collection of data, the researchers discovered that many of the genes encode proteins that fall outside standard classification schemes. Proteins grouped within their own unique kingdoms are turning up in other kingdoms as well—forcing the team to reconsider the evolutionary relationships of established kingdoms. “This project is revealing some of the biggest discoveries about the environment,” says Venter. (For more on these discoveries see the synopsis of the research articles [2].)

“If Darwin were alive today trying to do his experiments, he would not have been allowed to.”

## Untapped Diversity

**Figure 2:**
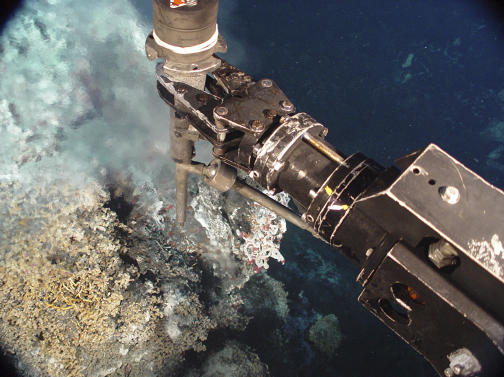
A Remotely Operated Platform Samples Vent Fluids from the Northeast Pacific Ocean (Photo: NOAA, http://oceanexplorer.noaa.gov)

The *Sorcerer II* probably captured only a tiny fraction of the genetic diversity out there, says Mitchell Sogin, Director of the Josephine Bay Paul Center in Comparative Molecular Biology and Evolution at the Marine Biological Laboratory in Woods Hole, Massachusetts. In August 2006, Sogin and his colleagues published a detailed analysis of variable stretches of ribosomal RNA collected from the marine microbial world (Figure 2) [3]. “We estimate there are at least 25,000 different kinds of microbes per litre of seawater,” says Sogin. “But I wouldn't be surprised if it turns out there are 100,000 or more.” A few of these microbes are common, and Venter will probably use them to recover complete gene sequences, he says. “The vast majority of low-abundance organisms are going undetected.”

Venter is more than aware that there's a lot more to be discovered, but for the moment the goal is to sequence as many genes, in their entirety, as possible from these ecologically rich environments. These data raise a host of intriguing questions: in particular, what is the structure and function of the novel proteins these genes encode, and what role do they play in the metabolism of these undescribed microbes? Just as Darwin's work drove a change in the way we see the world, so Venter is hoping these marine data will do the same in years to come.

## Legal Framework

But times have changed. In the 21st century, there are plenty of hurdles to clear before the collecting and describing of biodiversity—even microscopic biodiversity—can go ahead. The 1982 United Nations Convention on the Law of the Sea (UNCLOS) endowed coastal nations with the sovereign right to explore and exploit all resources within their “exclusive economic zone”—usually a body of water stretching 200 nautical miles out to sea [4]. Most coastal states exercise this right, granting permits to outsiders wanting to conduct research in their waters.

The 1992 Convention on Biological Diversity went on to set out some basic principles that might encourage sharing of benefits arising from genetic resources [5]. Where parties to the convention have got round to incorporating these principles into their own legislation, the result has been that anyone wishing to conduct research on these resources must agree to terms set by the host government.

Beyond national waters (with a few exceptions) are the “high seas”. Here, there is little regulation. According to UNCLOS, mineral resources on the deep seabed are considered the “common heritage of mankind”; this means that any benefits deriving from them should be shared with the international community. But when it comes to biological resources, just about anything goes.

## The Rise of Bioprospecting

In areas beyond national jurisdiction, there has been an increase in so-called bioprospecting, the search for and exploitation of commercially valuable compounds from genetic resources. In 2005, researchers at the United Nations University scoured patent office databases for inventions based on the genomic features of deep seabed organisms [6]. They found that private companies such as Roche, Diversa, and New England Biolabs are after patents on DNA polymerases developed from deep-sea thermophilic bacteria that promise to enhance the molecular biologist's expanding toolbox. Others like Sederma (based in France) and California Tan (based in the US) have used enzymes from similar microorganisms to develop skin products boasting UV- and heat-resistant properties.

**Figure 3:**
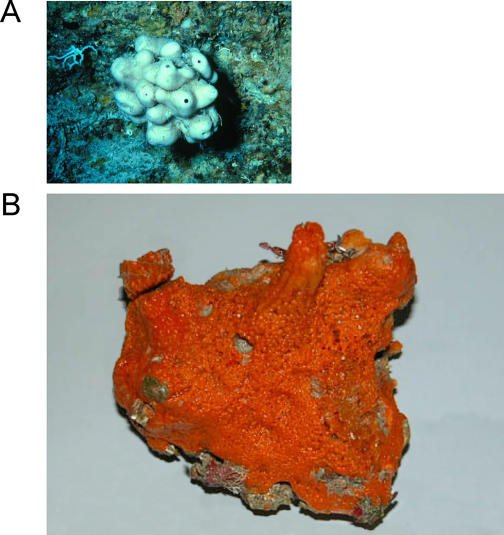
Marine Sponges That Have Generated Products with Anti-Cancer Promise (A) Discodermia dissoluta. (Photo: NOAA) (B) Forcepia triabilis. (Photo: T. Piper, NOAA)

There are plenty of not-for-profit organisations interested in the applications of discoveries from the deep. For example, Harbor Branch Oceanographic Institution, an oceanographic research and education institution based in Florida, is after compounds from marine organisms that might have biomedical potential. The institution has patents on, among others, potential anti-cancer agents derived from the marine sponges Discodermia dissoluta and Forcepia triabilis (Figure 3).

Deep-sea exploration, and the lengthy research and development that follows, is an expensive business. This means it's a realistic option for only the world's wealthiest nations. At least that's the concern being expressed by some developing countries that would like to see a piece of this action, says David Leary of the Centre for Environmental Law at Macquarie University in Sydney, Australia.

“No effort ever attempted to incorporate data from such vastly divergent sources to meet the needs of such a wide range of scientific interests.”

These countries are seeking a change to UNCLOS that requires biological resources to be treated in the same way as mineral resources and any benefits deriving from them to be shared with the wider community. But others fear tighter regulation of such activities will only stifle pure marine scientific research. The Philippines was one of the first countries to regulate access to its genetic resources, says Sam Johnston, an expert on international environmental law based in Melbourne, Australia, and a senior research fellow at the United Nations University Institute of Advanced Studies in Yokohama, Japan. “It basically closed down all research,” he says. “A lot of researchers around the world have found the red tape prohibitive.”

Finding a balance between the unregulated status quo and cumbersome controls over research on marine biodiversity is now the concern of a United Nations working group [7]. “Some countries see this as the early stage of negotiating a new UNCLOS,” says Leary. But, he warns, “this could take 10 or 15 years before we see a result.”

One compromise might be for coastal states to allow all research on their genetic resources with the proviso that exploitation of any commercial application is subject to further negotiation. Another possibility is for the patent system to take responsibility for seeing that benefits are shared fairly, only granting patents based on biological resources if a royalty is paid into a global commons trust fund.

## Ecological Impact

Whilst the UN goes in search of this kind of middle ground, both pure and applied research in the high seas continues apace—and this is cause for another concern. “There's a number of sites that are so popular that there's concern about the intensity of research,” says Leary. Repeated visits to the same deep-sea spot could not only result in unsustainable collection of some species and influence local hydrological and environmental conditions, but increase the likelihood that one person's experiment will influence that of another. So far, little thought has been devoted to this consequence of unregulated access, says Leary. “I haven't yet seen any clear scientific data on the extent of the environmental impact of bioprospecting or marine scientific research,” he says.

Clearly, the environmental impact of carrying off 150-odd barrels of seawater for analysis isn't something that Venter and his colleagues had to worry about. But navigating the complex legal territory was. “If Darwin were alive today trying to do his experiments, he would not have been allowed to,” says Venter.

At least, that is, without help from a lawyer. *Sorcerer II* collected samples in the waters of 17 coastal states and obtained all necessary permits, says Bob Friedman, Vice President for Environmental and Energy Policy at the J. Craig Venter Institute. Some countries required detailed agreements thrashing out how benefits deriving from these data would be shared. All of these are posted on the *Sorcerer II* Web site, says Friedman [8]. Most countries, however, have not decided how they might regulate access to their genetic resources, he says.

In addition to getting the paperwork in order, Venter encouraged collaboration with local scientists. What's more, the entire metagenomic database will be put in the public domain. The gene sequences should be of tremendous value to each of the countries involved, says Venter. In particular, it will help them monitor and manage the health of their marine ecosystems more effectively, he predicts. To ensure that this vast dataset will be available to all, the Gordon and Betty Moore Foundation has stumped up $24.5 million dollars for a seven-year project to design a new database to host it and new tools to interrogate it (Box 1).

**Box 1. Zooming in on CAMERA**

CAMERA is the convenient acronym for the cumbersomely named Community Cyberinfrastructure for Advanced Marine Microbial Ecology Research and Analysis. “This resource will focus on providing easy-to-use tools for uploading, downloading, searching, and analysis of genomic datasets,” says Paul Gilna, CAMERA's executive director, based at the California Institute for Telecom and Information Technology in La Jolla, California.

Researchers will also be able to clothe the bare genetic sequences in a wealth of other data, such as GPS coordinates and depth of collection, the water temperature, its oxygen content, salinity and pH. The site could well draw upon other resources that enrich these metadata, says Gilna. For example, satellite imagery associated with the sampling sites, and other data types, such as microscopy stills and high-definition video, could become important metadata that help researchers characterise the environments from which samples were taken.

Crucially, CAMERA will allow researchers to record the source of each genetic sequence. Many coastal countries now want a share of commercial applications that derive from their marine resources. Countries may be happy to see genetic sequences placed in CAMERA provided they are acknowledged and commercial exploitation of their sequence is not permitted without their consent.

But handling such immense datasets poses considerable technological challenges. The GOS database alone contains around 6 billion bases—the equivalent of two entire human genomes. And the number and size of this kind of database will only mushroom in coming years, making it necessary to develop high-speed optical networks, grid-based computing, and new visualisation technologies. “We are quickly approaching a ‘tipping point’,” says Gilna. “These datasets will start to follow exponential, rather than linear trends, much as was the case for DNA sequencing.”

Finally, there's the tricky task of satisfying all researchers who could benefit from this resource. “The scientific communities—from studies on biodiversity and biogeochemistry to evolution and genomes—have different interests, different data expectations, different vocabularies, and different levels of experience with using computational tools and databases,” says John Wooley, a pharmacologist at the University of California, San Diego, who is working on CAMERA. “Before metagenomics, no effort ever attempted to incorporate data from such vastly divergent sources to meet the needs of such a wide range of scientific interests.” For more on CAMERA, see the Community Page article by Seshadri et al. [13].

Yet, it seems, all these undertakings and assurances have not been enough to steer this expedition clear of controversy. In 2004, when the *Sorcerer II* dropped anchor just off Hiva Oa, an island in the Marquesas archipelago in the Pacific Ocean, tensions escalated. Although the plan to sample seawater around the islands had the backing of local French Polynesian authorities and scientists, the French government in Paris had other ideas, says Venter. “We were placed under house arrest.” Eventually, after a further round of intense negotiations, the *Sorcerer II* was allowed out of the harbour to collect its seawater samples and continue on its way.

Last year, a Canadian-based non-governmental organisation—the Action Group on Erosion, Technology and Concentration—dedicated to “the advancement of cultural and ecological diversity and human rights” labelled Venter a “biopirate”, accusing him of “flagrant disregard for national sovereignty over biodiversity” [9]. In several countries, there's real concern about how he managed his collecting, claims Pat Mooney, Executive Director of the group. Although the data are going into the public domain, it is laboratories like Venter's that are best placed to exploit it, he argues. “There's a handful of folk around the planet that can understand such stuff,” says Mooney.

Venter is adamant that this whole project is just pure, clean marine scientific research. Indeed, the *Sorcerer II* Web site explicitly states that “no intellectual property rights will be sought by the Venter Institute on these genomic sequence data” [10]. Venter sums up the goal of the project: “We were just trying to answer some basic questions about the diversity of microbes on the planet,” he says.

But, says environmental lawyer Johnston, the distinction between pure and applied research is becoming increasingly blurred. To illustrate this, he cites a strain of thermophilic Bacillus collected from Antarctica in the early 1980s as part of a study into the worldwide distribution and characteristics of such extremophiles. Years later, the same sample, taken out of storage and subjected to further study, turned out to contain a talented enzyme that has the promise to revolutionise DNA extraction for forensic analysis [11]. “The collector undertook the act in the purest form but ultimately the use of it has changed in the course of two decades,” says Johnston. “So much depends on the perspective at which you look at the issue.”

This means that there are likely to be several different takes on the same research. What for one person is pure marine scientific research can be another person's bioprospecting and yet another's biopiracy. There are very few cases where everyone agrees there has been outright theft of a biological resource and very few cases where everyone is happy there's been proper benefit sharing, says Johnston. “Even the best-designed programmes where there's enormous consultation with the local people have found it's difficult to get the right kind of consensus and buy-in,” he says [12].

So, keen as Venter might be to put the controversy of his human-genome-sequencing days behind him, this kind of research strays into unknown biological, legal, and ethical territory. And in this environment, allegations of biopiracy are almost inevitable. This, however, is unlikely to deter a man like Venter. “If it's in the Darwin school of biopiracy, then fine,” he says.

## Abbreviations

UNCLOS: United Nations Convention on the Law of the Sea
